# Fundamental Studies on the Use of Distributed Fibre Optical Sensing on Concrete and Reinforcing Bars

**DOI:** 10.3390/s21227643

**Published:** 2021-11-17

**Authors:** Tena Galkovski, Yasmin Lemcherreq, Jaime Mata-Falcón, Walter Kaufmann

**Affiliations:** Institute of Structural Engineering (IBK), Swiss Federal Institute of Technology Zurich (ETHZ), Stefano-Franscini-Platz 5, 8093 Zürich, Switzerland; lemcherreq@ibk.baug.ethz.ch (Y.L.); mata-falcon@ibk.baug.ethz.ch (J.M.-F.); kaufmann@ibk.baug.ethz.ch (W.K.)

**Keywords:** distributed fibre optical sensing, reinforcing steel, concrete compression

## Abstract

Distributed fibre optical sensing (DFOS) allows for quasi-continuous strain measurement in a broad range of gauge lengths and measurement frequencies. In particular, Rayleigh backscatter-based coherent optical frequency domain reflectometry has recently registered a significant application increase in structural concrete research and monitoring thanks to its numerous merits, such as high resolution and low invasiveness. However, it is not a plug-and-play technique. The quality of the acquired data depends highly on the choice of the fibre optical sensor and the methods of instrumentation and post-processing. Furthermore, its unprecedented resolution and sensitivity allow capturing local effects not well documented so far. This paper analyses the suitability of DFOS based on Rayleigh backscatter for reliably measuring strains and discusses the origin and structural relevance of local variations in the results. A series of experimental investigations are presented, comprising tensile tests on bare reinforcing bars and concrete compression tests. A critical analysis of the results leads to a best practice for applying DFOS to reinforcing bars and concrete, which establishes a basis for reliable, accurate measurements in structural concrete applications with bonded reinforcement.

## 1. Introduction

Distributed fibre optical sensing (DFOS) covers various technologies for strain measurements in a broad range of spatial resolution and measurement frequencies. In the field of geotechnics, DFOS has been used for quality control and structural health monitoring for several decades already [1,2,3,4]. Technological advances in Rayleigh backscatter-based coherent optical frequency domain reflectometry (c-OFDR) opened the way to use more affordable conventional telecommunication glass fibres for sensing, reaching high resolutions and frequencies [5,6,7]. Since then, c-OFDR has been increasingly used in structural concrete research [8,9,10] and monitoring [11]. The technology’s low invasiveness enabling instrumentation with minimum alteration of the actual structural behaviour and high long-term stability are core advantages besides the high measurement resolution and frequency.

The measurement reliability of this technology has been the focus of numerous investigations. DFOS strain measurements of bare reinforcing bars [12] and bare steel plates with varying cross-sectional geometries [13,14] have been compared to strain gauge measurements, digital image correlation (DIC) based strain measurements, FEM results and strains derived from equilibrium and constitutive material relations. The influence of fibre coating on the measurement quality has been explored, and a higher sensitivity of polyimide coated fibres than acrylate coated fibres has been observed. Brault and Hoult concluded from their investigations on reinforced concrete beams that polyimide coated fibres were less suitable for instrumenting reinforcing bars as they measured an unexpected jagged steel strain distribution [15]. On the other hand, Cantone et al. attributed this distribution to the high sensitivity of the sensing fibre and thus its capability to capture local discontinuities of the steel at ribs caused by mechanical interlocking with the surrounding concrete, leading to local tension and compression zones in the reinforcing steel around ribs [10].

Other research presents new horizons in data assessment and utilisation for structural concrete applications. The acquired data with c-OFDR is of unprecedented quality, thanks to the strain measurement’s low invasiveness and quasi-continuity. Monsberger and Lienhart showed the possibility to assess the curvature, bending moment and deflection distribution in grouted soil anchors monitored longitudinally with at least two fibres [3] and introduced a concept for distributed fibre optic shape sensing in concrete structures [16]. Instrumenting steel reinforcing bars with quasi-continuous fibre optical strain measurements also allows getting a deep insight into the crack behaviour of reinforced concrete members without the necessity of knowing the crack locations in advance [17,18,19,20]. DFOS directly measures the tension stiffening effect that could only be indirectly estimated until now for large-scale experiments with reasonable cost. With the DFOS data, characteristics such as the steel strain distribution (including mean and extreme values), crack spacing and crack widths can be assessed [21]. The normal and bond stress distribution can be estimated from the measured strains when knowing the bare reinforcing bar’s material law. Further, force equilibrium considerations at the cracks can be formulated for homogeneous structural elements to assess phenomena such as aggregate interlock in large-scale tests [22,23].

Regarding concrete instrumentation, fibre optical sensors (FOS) are often used to measure positive strains (tension) for crack assessment [24,25]. Concrete cracks are discontinuities with a discrete opening (i.e., have theoretically infinitely large strains). Therefore, when directly embedded in concrete or glued to its surface, the sensors need protection to avoid fibre rupture upon crack formation. The protection is typically provided by a robust jacket that smears the crack opening as a strain over a certain length, which varies from sensor to sensor depending on the jacket composition. While the measured strains lack direct physical meaning, they have been used to assess crack widths, e.g., through a mechanical transfer function [25] or by using a spring model calibrated for each FOS [24]. This approach is valid even for the case of overlapping strain peaks between neighbouring cracks. This instrumentation is very promising to gain insights into the behaviour of three-dimensional concrete structures, as crack widths inside the elements can be assessed at specific points by embedding FOS. However, most structures behave bi-dimensionally, and it is accurate enough to measure the crack behaviour just on the specimen’s surface. In such cases, it is more convenient to use other optical instrumentation techniques such as digital image correlation (DIC), which is easy to apply and allows for more detailed information (e.g., inspecting the entire crack pattern and measuring crack slip) when using automated approaches [26,27]. Therefore, concrete instrumentation is discussed in this paper exclusively for measuring compressive strains, which do have a direct physical meaning and are essential to understand the behaviour of structural concrete (concrete is typically assumed to carry only compressive loads).

The present work presents a concept for reliable strain measurements on concrete and steel reinforcing bars based on experimental investigations and fundamental concepts of the DFOS measurement technology. A best practice is proposed, including recommendations on instrumentation, sensing fibre type, data acquisition and data post-processing methods. In order to address contradictory findings in the literature on the suitability of polyimide and acrylate coated fibres for reinforcing bar instrumentation, both fibre coating types are discussed. The paper also explores limitations and drawbacks of DFOS: due to the high resolution of the measurement combined with a high sensitivity of the fibre optical sensor, local effects are captured and consequently, classic model assumptions (e.g., plane strain assumption, constitutive laws) can be applied to measured high-resolution strains only with limitations. Furthermore, a concept of measuring inelastic steel strains and in general strains almost up to the glass fibre’s ultimate strain is introduced, which so far has not been explored to the authors’ knowledge. The investigations are limited to short-term behaviour excluding thermal effects on the fibre optic measurement and influences from concrete shrinkage. These effects are discussed in a companion paper [28].

## 2. Research Significance

DFOS is used more frequently in structural concrete experimental investigations and monitoring. This work provides a comprehensive overview of the application of DFOS for concrete compression and reinforcing steel strain measurements. This overview aims at (i) facilitating the application for new users and (ii) ensuring the reliability of the measurements even for complex applications.

This paper focuses on material specific challenges regarding the interpretation and utilisation of the data. These challenges comprise measuring strains above the elastic range of the host (instrumented) material and inferring stresses from these strains using the constitutive law of the material. Another challenge is that classic approaches and models in structural concrete are not based on or derived from data with such a high resolution as provided by DFOS. Hence, this work discusses how to interpret and use this data properly. The paper also discusses examples of how the results of local strain variations might enhance the understanding of the behaviour of structural concrete subjected to uniaxial bending.

## 3. Fundamentals of c-OFDR Relevant for Reinforcing Steel and Concrete Instrumentation

**Figure 1:**
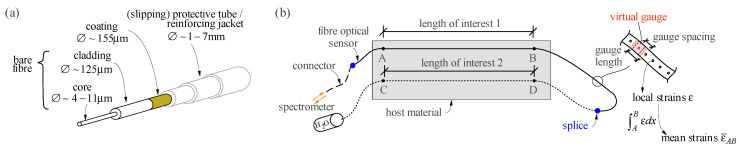
Fibre optical sensors: (**a**) composition of a single-mode fibre; (**b**) terminology used in the present paper.

This section introduces the fundamentals of c-OFDR and the underlying physics of light discussed in the context of structural concrete applications, focusing on single-mode fibres (Figure 1a). Understanding the fundamental concepts of the technology is essential to apply it correctly, benefit from it optimally, and understand and avoid potential problems.

### 3.1. Propagation of Light in Optical Sensors

A beam of light propagates through space in particular shapes. These shapes, also called modes, are limited to one mode in single-mode fibres by the physical restriction of the core diameter, which defines the mode field diameter and does not allow other modes than the first to form. In this way, modal dispersion of the light signal is avoided [1].

The refractive index of the core must be higher than the refractive index of the surrounding cladding to keep the signal inside the core. The smaller the difference of both refractive indices is, the higher is the angle of total reflection at the interface and the smaller the signal loss. Consequently, the refractive index of the cladding is chosen only slightly higher than that of the core. On the other hand, the material of the coating that protects the cladding from damage has a much higher refractive index to favour refraction to the outside and avoid the small portion of light refracted from the core into the cladding to be reflected back, causing signal dispersion [29].

### 3.2. Extrinsic and Intrinsic Attenuation

Signal losses are unavoidable and, in some cases, even desirable. One can distinguish between extrinsic and intrinsic attenuation. The former includes losses due to fibre bending and defects at the core-cladding interface. In such cases, light waves may be refracted and leave the core. Excessive bending, pinching of fibres and bad splices should be avoided since they cause increased local attenuation and distort the measurement. The sensing fibre termination also contributes to the extrinsic attenuation. At the fibre end, the signal is supposed to leave the fibre, i.e., perfect refraction should be pursued, as any reflected signal superimposes and distorts the backscatter, which is analysed for the strain measurement [29]. A simple method to maximise the light refraction at the fibre termination is discussed in Section 4.3.

Intrinsic attenuation includes the (back)scattering of light at imperfections and impurities of the glass. The kind of backscattered signal depends on the source light’s original wavelength and the size of the defect from which it is backscattered. It is distinguished between Rayleigh, Mie, Brillouin and Raman scattering. For coherent optical frequency domain reflectometry, the receiver evaluates the Rayleigh backscatter. It is generated at impurities of much smaller size than the wavelength and is linear, meaning it has the same wavelength as the incident light and does not exhibit a phase shift. Also, the attenuation loss due to Rayleigh backscatter is by far the strongest of the aforementioned types, achieving values around 0.2 dB/km at operating wavelengths of 1310–1550 nm [29].

### 3.3. c-OFDR: Rayleigh Scatter Based Distributed Fibre Optical Strain Sensing

In the simplest case of c-OFDR, a light source is coupled to a beam splitter. The light source sends out monochromatic light of low power and a linearly time-varying optical frequency ω. The beam splitter divides the signal into a reference signal and another signal that propagates into the coupled fibre optical sensor. Attenuation in the glass fibre creates a distinctive backscatter profile. The backscattered signal interferes with the coherent reference signal before the receiver registers it. Within the optical fibre at a certain location having a distance *x* from the spectrometer, the incident light is backscattered and hence travels for *t*_0_ *=* 2*x*/*v_g_*, where *v_g_* is the velocity of the wave inside the glass medium. During this time, the frequency *f* changes by *f = t*_0_ *d*ω/*dt*, where *d*ω/*dt* designates the change rate of the light’s optical frequency. The receiver hence detects a characteristic frequency *f* from a backscatter generated at the distance *x*. The frequency is proportional to *x* and can thus be transformed into the distance. A further important property is the backscatter amplitude: an imperfection in the glass fibre at distance *x* generates a backscattered signal of characteristic amplitude. A fast Fourier transformation of the detected signal delivers the amplitude-frequency plot, including frequency and specific amplitude information of each location *x* of the sensing fibre [6].

The spectrometer takes a reference measure (called reference state from now on) and correlates further measurements, i.e., registered backscatter signals, to it. Variations in temperature, deformations, but also humidity cause a change in the backscattered signal. When an optical fibre is elongated, the amplitudes of a signal backscattered at the impurities within the deformed section do not change in consequence, but the transmission time, and hence the frequency*,* change in proportion with the position. Hence, changes in the position of the impurities can be assessed using the optical information [7] and processed to determine strains.

An optical distributed sensor interrogator ODiSI-6104 supplied by Luna Innovations Incorporated was the spectrometer used in this work. A fibre optical sensor, which might be composed of multiple fibre segments spliced together, is spliced to a connector and then switched to the spectrometer. The device generates a reference state of the fibre optical sensor (also called key), from which it is able to measure strains up to a range of ±15,000 μm/m with an accuracy of ±1 μm/m [30]. Since this measuring range is lower than the failure strain of reinforcing steel bars and concrete, the measurement of plastic reinforcement strains and non-linear concrete strains typically requires defining several reference states during an experiment, as will be further discussed in Section 4.3 and Section 5.1. The spectrometer does not assess strains for every backscattered signal it registers, but bundles the information over defined virtual gauges. The strain of each virtual gage ε will be referred to as local strain, while ${\bar{\varepsilon }}_{AB}$ denotes the averaging of local strains over a distance $\bar{AB}$. The gauge spacing (distance between the centres of two adjacent virtual gauges) and the gauge length are set equal (“gauge pitch”) in this device. Figure 1b introduces this and other terminology used in the paper. The suppliers advise using the device’s minimum gauge pitch (0.65 mm) to achieve the best data quality. All experiments presented in this paper used this recommended value. The spectrometer measures at a maximum frequency of 62.5 Hz with this gauge pitch. The frequency decreases with increasing measurement length (up to 100 m per channel), but typically lies above 1 Hz.

### 3.4. Sensing Fibre Composition

Single-mode fibres are used in Rayleigh based distributed fibre optical strain sensing. The diameter of the fibre core ranges from 4 to 11 μm, and usually is 9 μm (see Figure 1a). The cladding, whose function is to keep the signal inside the core, usually has 125 μm in diameter. Within this work, the core and the cladding are referred to as the bare fibre. A coating is applied to protect the bare, brittle fibre. The nature of this coating essentially dictates the sensor’s sensitivity. In case of mechanical bond between coating and cladding (e.g., acrylate), a significant amount of slip occurs, causing a smoothening of the measured strains. In contrast, with a chemically bonded coating (e.g., polyimide), the sensor has a much higher sensitivity and a significantly shorter activation length since slip is minimised. This type of fibres is commonly used to instrument plane surfaces such as steel using an appropriate adhesive.

Additionally, some sensors are equipped with further protection, such as a slipping tube that needs to be removed before mounting. Such a tube may also be bonded and can then only be removed by mechanical impact with special pliers. For other types of sensors, the bare fibre is placed inside a tight metal casing and is only activated through friction and encased by a rather rigid and robust jacket consisting of polyimide, occasionally also reinforced with metal inlays. These jackets vary in thickness and should be chosen appropriately since they significantly reduce the sensor’s sensitivity: local strains are smeared over a significant length. The more robust sensors are mainly used for geotechnical and tunnelling applications, whereas others, usually with up to 3 mm in diameter, are suitable to be directly embedded in concrete.

## 4. Best Practice for Strain Measurements of Concrete and Steel Using DFOS

**Figure 2:**
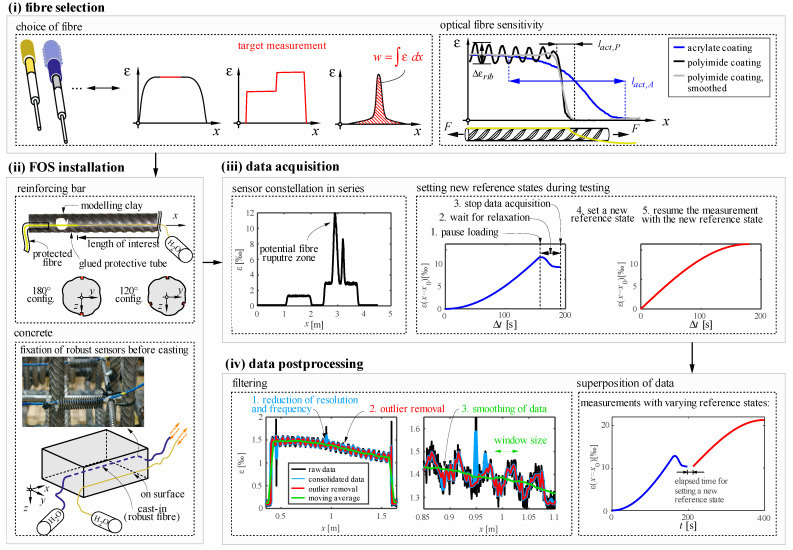
Workflow for fibre optical strain measurement on steel and concrete comprising the following steps: (**i**) Fibre selection, (**ii**) installation on the host material, (**iii**), data acquisition and (**iv**) data post-processing.

This section introduces the established practices for the application of DFOS on reinforced concrete at the Chair of Concrete Structures and Bridge Design at ETH Zurich. The best practice comprises (i) the fibre selection, (ii) the installation of the sensing fibre, (iii) data acquisition, (iv) post-processing of the raw data, and (v) the assessment of errors and bias. Figure 2 presents an overview of the workflow steps, which are described in detail in Section 4.1, Section 4.2, Section 4.3, Section 4.4 and Section 4.5. This best practice reflects our experience with DFOS since our first use back in 2015. It comprises exclusively the strain measurement. The details on how to derive additional properties from the data are discussed in a companion paper [28].

The application of fibre optical instrumentation aims at capturing the host material behaviour with minimum alteration. A stiff bond between the sensor and the host material and a precise alignment are therefore essential. In structural concrete applications, the glass fibres can be mounted on the concrete surface, inside the concrete or on the reinforcement. In either case, the instrumentation is able to measure compressive and tensile strains. However, due to the nature of reinforced concrete, where tension is mainly carried by the reinforcement and compression by the concrete, this work focuses on the measurement of concrete compressive strains and tensile strains in ribbed reinforcing steel bars.

### 4.1. Selection and Installation of Sensing Fibres for Reinforcing Steel Instrumentation

Ribbed reinforcing steel bars are typically instrumented through an optical fibre glued onto the bar surface. The reinforcement deformations are transferred through several layers, starting from the bar (i.e., host material) to the glue, coating, cladding and finally, the fibre core, where the measurement takes place. Slip between these layers and the stiffness of each layer governs the difference between the actual strains in the host material and the measured strains in the fibre core. To minimise this undesirable difference, the thickness of the glue layer needs to be kept small, and the coating of the fibre should have a small thickness, high stiffness and little slip (i.e., single-mode fibres with a thin coating are most suitable for this purpose). Using this type of fibre is essential when sharp strain gradients occur (e.g., when measuring plastic strains for steels with distinct yield plateaus, see Section 5.1). However, less sensitive sensors might also be used if relatively constant or linear strain profiles are of interest or even when the measured strains are to be integrated. Hence, the target strain profile that should be measured decides whether a specific sensor is appropriate or not (Figure 2i).

Even when using fibres with a thin coating, the potential slip and the coating stiffness are very dependent on the coating material. Acrylate coatings are less stiff and can be removed by mechanical impact alone. When deformed, the glass fibre is prone to slip inside the coating. Polyimide coating, on the other hand, is bonded chemically to the glass (i.e., can only be removed by applying acid or heat impact) and shows very little slip. Hence, polyamide coated fibres should be used when local strain gradients are to be captured. The different behaviour of fibres with both coatings is shown on the right side of Figure 2i. This figure shows strain measurements on a reinforcing bar using acrylate and polyimide coated fibre optical sensors, respectively, installed inside the same groove. A significant difference can be observed in the activation length (i.e., the length over which the measurement smooths a strain discontinuity) between both coatings. Moreover, the polyimide coated fibre is sensitive enough to capture even local strain variations caused by the ribs. The causes for these local variations will be discussed in Section 5.1. These variations are usually irrelevant for the global structural behaviour and can be removed in the post-processing phase (it is recommended to average the strains over a length equal to the rib spacing, see the grey line on the right side of Figure 2i). Whereas sensors with acrylate coating do not capture undesired local effects, they also miss local maxima or minima if these occur within a section smaller than twice the fibre activation length.

The fibre optical sensors on reinforcing steel can be installed by gluing the bare glass fibre alongside the reinforcement’s ridge or preferably inside a groove (Figure 2ii) for better protection. The dimension of the groove should be kept as small as possible but be able to accommodate a protective tube at its ends. The groove should not go through the ribs and be machined without causing high temperatures (e.g., planed) to minimise the influence on the behaviour of the reinforcing bar. After removing oil and dirt residues in the groove, the fibre is placed inside with protective tubes at the ends (see details in Figure 2ii). Modelling clay is used to fix the tubes and seal the groove. The fibre is straightened by applying tension using weights and fixed, e.g., with magnets. The groove is finally filled with epoxy.

It is recommended to instrument the reinforcing bars with at least two opposite longitudinal fibres (Figure 2ii) to account for potential bending effects. The average of the measured strains provides the mean strain of the reinforcing bar if the strain distribution is assumed to remain plane. Three fibres are, however, needed if the strain plane of the cross-sections is of interest (e.g., to measure bending effects).

While the small grooves have a negligible influence on the behaviour of the reinforcing bars in most cases, the cross-sectional loss might be significant for bars of small diameters. The material properties of reinforcing bars with distinct microstructure (e.g., quenched and self-tempered bars) might also be slightly modified, since the groove removes part of the outer (martensitic) layer of the bar [31]. Therefore, whenever stresses or forces are to be derived from the measured strains in bars of small diameters, it is good practice to determine the steel’s constitutive law on grooved samples [17].

### 4.2. Selection and Installation of Sensing Fibres for Concrete under Compression

The targeted measurement is also essential for choosing a suitable sensor type when instrumenting concrete. The sensor sensitivity should be appropriate for the intended application (i.e., do not smear strain peaks or gradients excessively). Limiting the sensor diameter and conducting tests to check its sensitivity in advance is recommended, particularly for jacketed sensors.

The optical fibre can be cast-in or glued on the concrete surface to measure compressive strains (see Figure 2ii). It is essential that the sensors are tensioned and fixed before casting or gluing to ensure their straightness, following similar operations as outlined in Section 4.1 for reinforcing bars or for robust sensors with springs as illustrated by Figure 2ii. Robust sensors need to be used for cast-in applications to withstand the impact of aggregates during casting. The market provides specific jacketed sensors suitable for this use. Such sensors consist of a single-mode sensing fibre protected by one or several coating layers (see Figure 1b). Removing the jackets to splice a connector or another sensor to the optical fibre is cumbersome. The splice is often a weak point that should be protected adequately. When directly glued on the concrete surface, fibres without jacketing and much higher sensitivity can be used (e.g., polyimide or acrylate coated fibres already described for instrumenting reinforcing bars, see Section 4.1). The performance of sensors with and without jacketing will be compared in Section 5.2.

### 4.3. Data Acquisition

In Section 3.2, the importance of low extrinsic attenuation for the quality of the measurement was emphasised. The fibre termination requires some preparation to favour refraction and keep disturbing attenuation low. It is recommended to cut it at a 45° angle and place it into water. Because water has a similar refraction index as the glass fibre core, the incident light is mainly refracted instead of reflected. Alternatively, a coreless glass fibre can be spliced at the end. While sensors with a suitable termination can also be acquired, the authors recommend these custom-made solutions given their proven reliability, simplicity and low cost.

Multiple fibre optical sensors can be spliced to be interrogated together. If a significantly different strain measurement is expected in each sensor, the sensor order should be selected strategically. Fibre sections with the highest expected strains should be placed at the end (see Figure 2iii) to minimise the noise in the remaining sensing length. In case of a fibre rupture, this also allows continuing to measure strains up to the fracture location.

**Figure 3:**
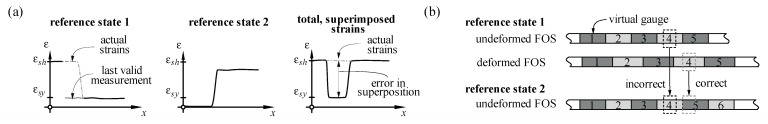
Potential issues in DFOS measurements with multiple reference states: (**a**) Loss of correlation when measuring steel with a yield plateau; (**b**) potential incorrect superposition when setting a new reference state that defines new virtual gauges with a different location.

The correlation is often lost for (i) high strain gradients, (ii) strains close to the measurement range of the spectrometer and (iii) high extrinsic losses (see Section 3.2). Ideally, before correlation is lost, a new reference state should be set. From this new reference state, another ±15,000 μm/m can be measured. Absolute deformations can be computed in post-processing by superposing the data of the different reference strains. To avoid biases in this superposition, the following data acquisition steps are recommended:

- Set a new reference state before losses in the measurement take place: imputing lost data with the last correlated value or by linear interpolation of neighbours is prone to errors in regions with high strain gradients. This is illustrated in Figure 3a for a measurement of a reinforcing steel bar with a distinct yield plateau.
- DFOS acquisition needs to be stopped to generate a new reference state (Figure 2iii). To minimise uncaptured deformations while the acquisitions is stopped, one should pause the loading and wait for a significant part of relaxation to be completed before starting the process of generating a new reference key.
- Superpose the strains of virtual gauges at similar locations: While some spectrometers keep the original sensors virtual gauges when generating a new reference state, others generate an entirely new sensor (i.e., the deformed fibre optical sensor is divided into new virtual gauges with the gauge pitch set in the first state). The latter is the case for the spectrometer used in this work, in which the location and number of gauges might change in each reference state. Superposing data of gauges with the same number might be inaccurate (as shown in Figure 3b) for large sensing lengths, high strain levels and small gauge pitches.

### 4.4. Data Post-Processing

**Figure 4:**
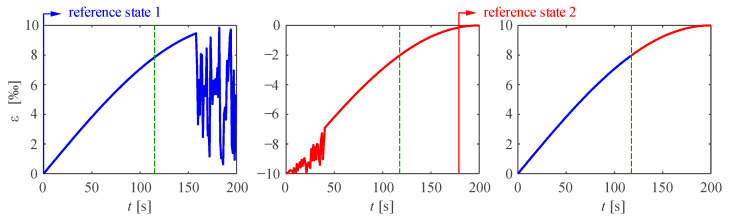
Restoring noisy data with a spectrometer maintaining the same virtual gauges in all reference states and a measurement correlation in the post-processing.

While the device used in this work generates strain measurements directly during data acquisition, other spectrometers (e.g., the ODiSI-A supplied by Luna Innovations Incorporated) produce primary data, which must be post-processed. For spectrometers setting identical virtual gauges for all reference states (e.g., the ODiSI-A device), performing the correlation in the post-processing allows minimising the measurement noise. Since any measurement can be correlated to any reference state, the optimum correlation is chosen for each measurement range, as shown in Figure 4.

Once the raw strain data is obtained, some post-processing steps are recommended (Figure 2iv). The main steps are (1) reduction of the data to the length(s) of interest only, and downsampling the data (resolution and frequency) to suitable values (data consolidation), (2) filtering the data, starting with outlier removal and if necessary smoothening the data, (3) superposition of data measured with various reference states, (4) deriving other values from the data and (5) plausibility checks. The details of each step are described in the following sections.

#### 4.4.1. Consolidation

First, measurements at locations outside the length of interest should be removed to reduce the amount of data. Consolidation of data in space and/or time is further advised. For quasi-static structural concrete experiments, frequencies above 1 Hz and resolutions of 0.65 mm are excessive. Within the consolidation process, larger virtual gauge lengths and smaller measurement frequencies are generated, e.g., by averaging or interpolating the original data. This allows reducing the noise of the data, as can be seen in the filtering part of Figure 2iv and thinning out data points in which the correlation was lost. In the case of reinforcing bars, it is recommended to increase the (apparent) gauge spacing to a length equal to the rib spacing to mitigate the local effects due to the ribs discussed in Section 4.1. For concrete measurements, a value equal to the maximum aggregate size is recommended. The data is either completely consolidated to such a gauge spacing or just slightly reduced and filtered to reach the targeted apparent gauge spacing in the next step.

#### 4.4.2. Filtering

When filtering data, a good practice is to remove outliers in the first step. The median *m* and standard deviation σ can be determined within a given window around one particular result. Suppose the result deviates excessively, e.g., above ±*n*σ from *m* (in this study, a value *n* = 3 was considered). In that case, it is identified as an outlier. Such an outlier filter should be applied over the space and time domains. Figure 2iv presents an example of data outlier removal (red curve). The result is highly sensitive to the chosen window size and multiplier *n*. These parameters should be set carefully. Actual discontinuities, e.g., at the onset of concrete cracking, should not be removed. The outlier removal can be performed either before or after the data consolidation. As an alternative, Fischer et al. proposed to use the spectral shift quality for outlier detection and removal, which works well [19].

Filtering should be done as little as possible but as much as necessary and be clearly documented and reported. Excessive smoothening can shift local minima and maxima or bias their values similar to the low sensitivity of the sensor. The need for data smoothening depends on the intended use of the obtained strains. If they are integrated or averaged, there is no need to apply additional filters. This is also often the case when stresses are to be derived by considering a certain material constitutive law. However, data smoothening should still be applied if local effects such as the influence of ribs in reinforcing bars have not been totally removed within the consolidation phase.

#### 4.4.3. Superposition

Relative strains of different reference steps need to be superimposed to get absolute strains. While the raw data could be superimposed, it is recommended to superimpose the filtered data for the sake of computational efficiency and to avoid summing up the noise of different measurements. If the last measurement of a reference step has correlation losses or noise that cannot be filtered out, the new reference state was set too late. In such cases, the only solution is either taking the last valid measurement or interpolating using neighbouring results. However, this practice should be applied with caution and documented because it is prone to errors, as already discussed in Section 4.3.

### 4.5. Further Steps and Assessment of Errors and Bias

The post-processed strain data can be used to derive other quantities relevant for the structural behaviour. The derivation of stresses will be discussed in Section 6, while the calculation of other quantities such as bond shear stresses, slip or crack spacings are presented in a companion paper [28]. The comparison of these derived magnitudes to the results of other instrumentation constitutes a good plausibility check to assess potential measurement errors. Some possibilities are listed in the following.

The measured local strains can be directly compared to strain gauge measurements or full-field digital image correlation (DIC). By integrating the DFOS strains, deformations, inclinations, or curvatures can be calculated, which can be compared to measurements from LVDTs, inclinometers or DIC. Stresses derived from the measured strains can be checked against engineering stresses determined from applied loads and geometrical information. This requires knowing precisely the constitutive law of the host material, which is particularly challenging for repeated loading and unloading cycles in the plastic range of the materials (see Section 6).

## 5. Basic Investigations of Strain Sensing on Reinforcing Steel and Concrete

This section presents experimental data used to establish and support the best practice introduced in Section 4. The study comprises tests on (i) bare reinforcing bars made of quenched, self-tempered steel and hot-rolled, cold-worked steel subjected to uniaxial tensile loading and (ii) the concrete compression zone of a beam subjected to a constant bending moment. Table 1 summarises all specimens. The used spectrometer and main processing parameters were described in Section 4.

### 5.1. Measurement of Reinforcing Steel Strains

When a reinforcing bar is deformed longitudinally, the resulting strains and stresses are not constant due to (i) the irregular ribbed surface and (ii) the non-homogeneous material properties over the cross-section. The ribs induce slight variations in cross-sectional area along the bar axis and discontinuities that disturb the strain (stress) field and cause strain (stress) concentrations. Local strains are higher than the average strains (e.g., measured by an LVDT) in the inter-rib areas and lower at the ribs. In Section 5.1.3, these phenomena are analysed for a bare reinforcing bar to allow for a correct interpretation of local strain variations captured by DFOS.

**Figure 5:**
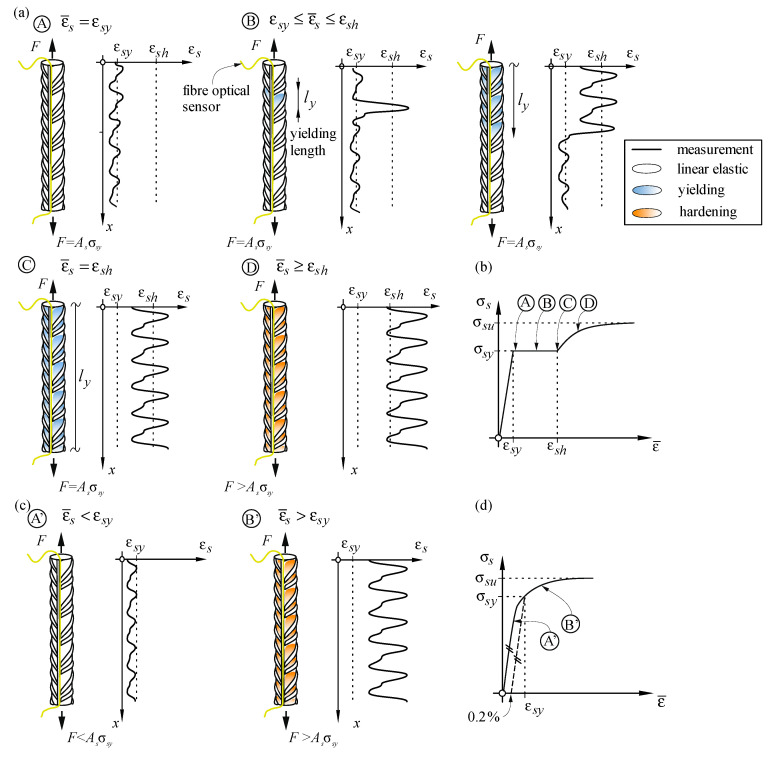
Deformation behaviour of ribbed reinforcing steel bars: (**a**) DFOS strain profiles of QST steel bar at deformation steps A (start of yielding), B (yielding), C (end of yielding) and D (hardening); (**b**) constitutive material law of QST steel; (**c**) DFOS strain profiles of CW steel bar at deformation steps A’ (linear elastic) and B’ (plastic) and (**d**) constitutive material law of the CW steel bar (QST refers to quenched and self-tempered steel, while CW refers to hot-rolled and cold-worked steel).

The production process influences the constitutive law of reinforcing steel, setting various challenges for DFOS instrumentation. Nowadays, two production processes of reinforcing bars are the most frequent. The first one consists of hot-rolled reinforcement that is cold-worked after slowly cooling down. Such cold-worked steel (CW) has a homogenous microstructure (ferrite and perlite), lacks a yielding plateau and has a lower strain at ultimate strength than it would have without being cold-worked. In the second production process, steel is quenched, i.e., cooled down quickly with water (or air in some cases), after being hot-rolled, in such a way that the core remains hot enough to temper the outer layer after cooling. As a result, the core is a homogenous ferrite and perlite mixture with high ductility, while a martensitic external layer with higher strength and a bainite transition zone between the two [31] form. Unless coiled, quenched and self-tempered (QST) reinforcing steel exhibits a distinct yield plateau after the elastic phase, followed by hardening. Figure 5 shows the behaviour of both reinforcing steel types qualitatively.

While CW steel bars deform homogeneously throughout both the elastic and the inelastic phase (Figure 5c,d), QST steel starts yielding locally (Figure 5a,b). The yielding front of QST bars, known as Lüders bands propagates through the reinforcing bar (state B) until the entire bar yields (state C). Hardening sets in at this point, and strains increase homogeneously over the bar length again (state D). During the yielding phase (state B), a discontinuity from ε*_sy_* to ε*_sh_* is produced at the Lüders band fronts. Since this strain jump is typically above the measuring range of the spectrometer, measuring QST bars in the plastic range with DFOS is cumbersome. Measurements for both types of reinforcing steel are presented and discussed in Section 5.1.1 and Section 5.1.2.

#### 5.1.1. Cold-Worked Reinforcing Steel

**Figure 6:**
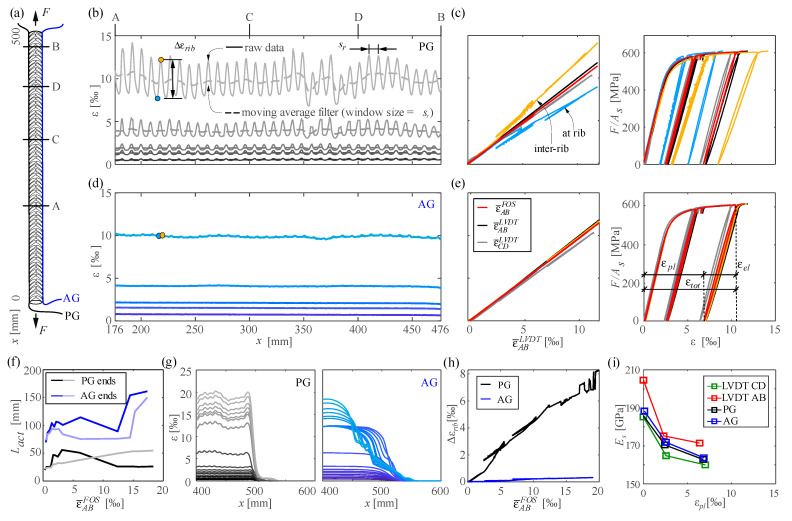
Cold-worked reinforcing bar cw01 tested under pure tension: (**a**) Test setup with polyimide (PG) and acrylate coated fibres (AG); (**b**,**d**) raw and filtered strain distributions within $\bar{AB}$ at selected load steps; (**c**,**e**) comparison of local strains at rib peaks and valleys with mean strains by LVDTs and DFOS, and resultant stress–strain behaviour; (**f**) activation length at both ends of PG and AG fibres; (**g**) filtered strain distributions at the right end at various load steps; (**h**) strain difference between rib valley and peak and (**i**) secant modulus as a function of plastic strains.

**Figure A1:**
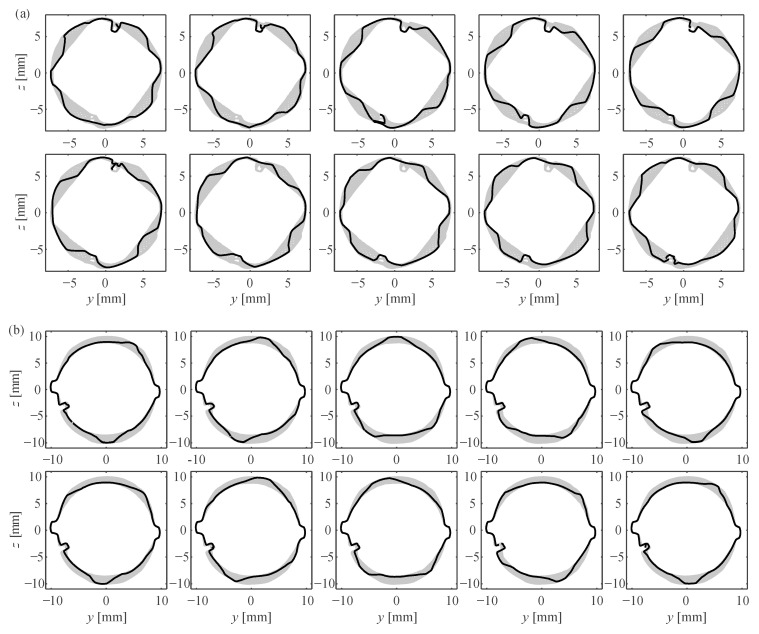
Variation of cross-sectional geometry showed on ten equally spaced cross-sections within a repeating rib pattern: (**a**) specimen cw01 ∅14 and (**b**) specimen cw03 ∅18 mm.

The particularities of instrumenting cold-worked reinforcing steel are studied by means of specimen cw01. This test and specimen cw03 were conducted in the framework of a Master’s Project Thesis [32]. The bar has a diameter of 14 mm, and its cross-sections determined by a surface scan are shown in Appendix A (Figure A1a). The test setup and instrumentation are shown in Figure 6a. One side was instrumented with a polyimide coated fibre (PG) and the opposite side with an acrylate coated fibre (AG), both glued inside a 1 × 1 mm groove passing directly through the ribs. Additionally, two LVDTs were placed over $\bar{AB}$ and $\bar{CD}$. The bar was tested under direct tension. The bar was unloaded and reloaded at approximately the following average strains levels (i) 1.2‰, i.e., 60% of the yield strain, (ii) 5.5‰ and (iii) 10.5‰. Figure 6 presents an overview of the results for both fibre types. While the plots (b)–(e) and (h)–(i) focus on the DFOS measurements over the distance of the longer LVDT (i.e., $\bar{AB}$), the plots (f) and (g) present the results over the entire sensing length to analyse the fibre activation length (*L_act_*).

The local strain measurement with the polyimide coated fibre (Figure 6b) confirm the qualitative behaviour presented in Figure 5c,d. Local extreme values are visible in the raw data at a spacing similar to the rib spacing. The strains in the inter-rib area (yellow) are much higher than at the ribs (blue). Figure 6h shows that their difference increases almost linearly with the applied strain level and amounts to around 40% of the mean strain. When using a moving average filter of a window size corresponding to the rib spacing (13 × 0.65 mm in this case) as recommended in Section 4.4.1 and Section 4.4.2 the influence of the ribs can be cancelled out while preserving other relevant local information. For instance, the strain disturbances visible after filtering in Figure 6g coincide with visible defects on the bar caused by clamping during the slicing of the grooves. This clamping might have caused a local cold forming of the steel, which affects the steel behaviour locally. Besides local observations, the strain results can also be averaged to capture the mean behaviour over a certain distance (e.g., to derive material constitutive relationships, typically derived from mean strain results). Figure 5c shows that the average strains derived from the local DFOS information (${\bar{\varepsilon }}^{FOS}$) are in excellent agreement with the average strains measured by LVDTs (${\bar{\varepsilon }}^{LVDT}$).

The acrylate coated fibre captures the fluctuations caused by the ribs only slightly at high deformations (Figure 6d,h). Local strains (blue and yellow), mean DFOS strains and the engineering strains from the LVDT coincide very well in this case (Figure 6e). The effect of the clamping defects measured by the polyimide fibre is almost invisible due to the lower sensitivity of the fibre. The different sensitivity of the used fibres can be better quantified when comparing their activation lengths (Figure 6f,g). The activation length was quantified as the length with a strain gradient higher than 50 μm/m^2^ (filtered strains were used to avoid the local influence of the ribs). The activation length of the AG sensor in the elastic range was 70 mm, around five times larger than the activation length of the PG sensor. For both fibres, the activation length increases with the imposed strain level. It should be noted that the right end of the AG fibre slipped through the coating at a strain level of only 12‰. These observations support the recommendation of using polyimide coated fibres when instrumenting reinforcing steel bars. While the potential drawbacks of highly sensitive polyimide fibres can be compensated in the post-processing phase, acrylate coated fibres might fail to capture relevant information.

The use of DFOS to evaluate the stiffness of the reinforcement is shown in Figure 6i. To this end, the secant modulus of each of the three unloading and reloading branches was quantified using the available sensors (average local fibre optical strains over the distance $\bar{AB}$ were used). All sensors show consistently a decrease in stiffness of the reinforcing bar with increasing plastic deformations (for a total strain of 10.5‰, the stiffness of the unloading branch is about 15% lower than stiffness in the linear elastic range). The use of true strains and stresses, accounting for the reduced cross-section due to yielding, leads to very similar results to the used engineering strains and stresses.

**Figure 7:**
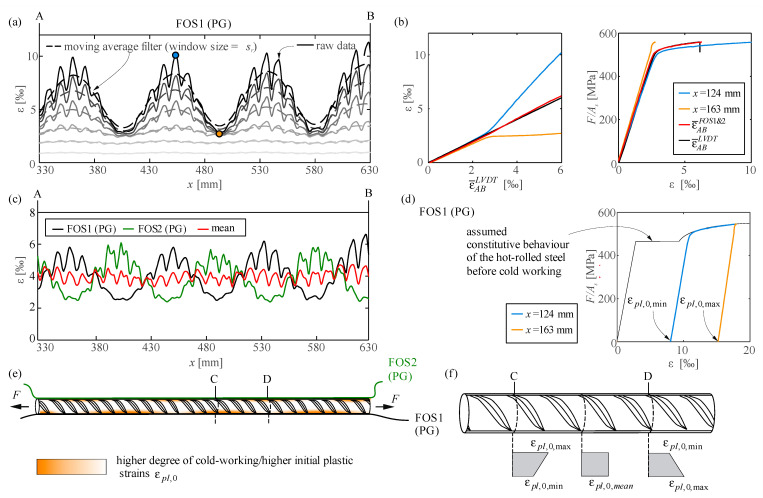
Cold-worked reinforcing bar cw02 tested under direct tension: (**a**) Strain distribution of FOS1 at selected load steps; (**b**) comparison of local strains at marked points to mean strains of LVDT and FOS over $\bar{AB}$, and resultant stress–strain behaviour; (**c**) strains distributions of FOS1&2 and their average for a 4‰ mean; (**d**) qualitative explanation of different local stress–strain behaviour with initial strains; (**e**) uneven cold-working degree and (**f**) qualitative distribution of local initial strains (ε*_pl_*_,0_).

A varying cold-working degree that leads to inhomogeneous strains (discussed for the clamping defects in cw01) may as well be generated during the fabrication of the reinforcement if the tolerances of the cold-working process result in a variable degree of stretching along the bar. Such is presumably the case of the reinforcing bar cw02 shown in Figure 7. The bar was instrumented with two polyimide-coated glass fibres (FOS1 and FOS2) glued in grooves in a 180° configuration and an LVDT. The results in Figure 7a,c show that FOS captured a very distinct wave-like strain distribution after the onset of yielding. The wavelength is about 90 mm (i.e., not related to the rib spacing of around 12 mm). Figure 7b shows that the difference between the maximum and the minimum strains increases linearly with the applied strain level. The measured local constitutive behaviour (Figure 7b) shows that the regions with lower strains remain elastic whereas other parts reach strains above 10‰ (i.e., not the entire bar yields). The two fibre sensors measure similar strain distributions but opposite phases (Figure 7c). Hence, their average results in fairly constant strains (see red series in Figure 7c, in which only local peaks due to ribs are present).

The observed strain distributions in cw02 are likely to be the result of uneven cold working. Three reinforcing bars of the same batch were tested, and all of them showed this particular strain distribution, which differs from the typical constant strain distribution of cold-worked reinforcing steel bars. Figure 7d–f present a qualitative explanation for the observed deformation behaviour. A defect in the cold working process might have introduced a periodical asymmetry in the stretching of the hot-rolled steel. Plastic hinges of changing sign have formed. This would have led to variable initial strains (ε*_pl_*_,0_) in the bar and even within sections, despite they are expected to be constant in all cross-sections on average (Figure 7f). Variable initial strains ε*_pl_*_,0_ result in a different constitutive behaviour at each location. The regions with the highest initial plastic deformation (ε*_pl_*_,0,max_) have the largest yield strength but the lowest deformation capacity. Whereas yielding starts in those regions of the reinforcing bars with the lowest initial strains (ε*_pl_*_,0,min_), the areas with minimum deformation capacity (i.e., with highest initial strains and minimum measured strains) might fail first.

This example highlights the necessity of instrumenting reinforcing bars with several sensors to capture their overall behaviour. While the observed variable strains within a cross-section might not be relevant in many cases, they are of interest when studying structures in which the local behaviour is decisive (e.g., concrete structures with corroded reinforcing bars [33] or reinforcing bars with low ductility).

#### 5.1.2. Quenched and Self-Tempered Steel

**Figure 8:**
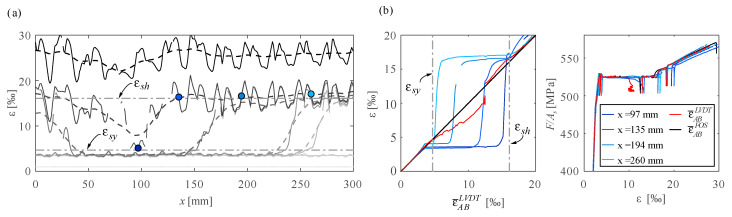
Quenched and self-tempered reinforcing bar qst01 tested under direct tension and instrumented with polyimide coated fibre glued inside a groove: (**a**) DFOS strain distribution at selected strain levels and (**b**) comparison of local DFOS strains at marked points with mean strains by LVDTs and DFOS, and resultant stress-strain behaviour.

This section discusses the results of DFOS on quenched and self-tempered steel (QST) bars by means of specimen qst01. In this test, a bar of diameter 20 mm was tested monotonically in direct tension. The instrumentation consisted of fibre optics—a polyimide coated fibre glued inside a groove with epoxy (PG)—and an LVDT installed over a distance of 300 mm. The extension of the yield plateau ($\varepsilon_{sh}-\varepsilon_{sy}$) of the steel was exceptionally small in this bar, which allowed measuring the yielding phase without losing correlation and data while setting a new reference. $\varepsilon_{sh}-\varepsilon_{sy}$ typically exceeds the measurement range of the device (±15‰), leading to correlation and data losses, as discussed in Section 4.3. Figure 8a,b show how local strains jump suddenly from the yielding strain $\varepsilon_{sy}$ to the hardening strain $\varepsilon_{sh}$, as already discussed in Section 5.1 (states A to C in Figure 5a). It should be noted that no filtering was applied in this case to the fibre optic strain results to see more distinctly the Lüders bands front.

**Figure 9:**
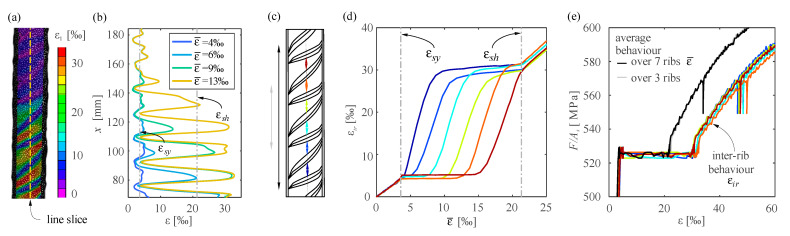
Progression of Lüders bands captured by DIC shown on specimen qst02: (**a**) strain field during yielding ($\bar{\varepsilon }=13‰$); (**b**) strain distribution along the line slice in (**a**) at selected strain levels; (**c**) location of virtual gauges; (**d**) comparison of inter rib strains to mean strains over seven ribs, and (**e**) measured stress–strain behaviour with virtual gauges of different length and location.

The Lüders bands progression in quenched and self-tempered reinforcing steel can also be measured through digital image correlation (DIC). Provided that a sufficient resolution and frequency are used, DIC can track plastic strains in bare bars regardless of the length of the yielding plateau, as shown in the following for test qst02. In this test, a bar of 26 mm in diameter was tested monotonically in direct tension as part of a larger experimental campaign [34]. The bar was speckled manually with a speckle size of 0.7 mm. The 3D-DIC system was composed of two Allied Vision Prosilica GT6600 cameras with a resolution of 28.8 MPx and Quioptic Rodagon 80 mm lenses with a baseline of 264 mm, which resulted in an average scale of 0.05 mm/px. The correlation was carried out with the software VIC-3D (Correlated solutions inc. [35]) using a subset size of 53 px, step size of 7 px and a strain filter size of 9. The local strain results in Figure 9a,b at four instances during yielding show the Lüders bands propagation from the bottom to the top of the bar. As expected, the load stayed constant during the yielding phase (Figure 9e). The local jumps in the strains reached almost 30‰. Hence, measuring the hardening phase of this bar with DFOS would have been challenging. These jumps are not only due to the yielding process but also due to the influence of the ribs. An analysis of average strains of the bar and at the inter-rib (see the location of DIC gauges in Figure 9c) shows that the inter-rib strains are around 50% larger than the average strains (Figure 9b). This difference is higher than for cold-worked reinforcement (see Figure 6c, inter-rib strains are around 20% larger than mean strains).

#### 5.1.3. Influence of Ribs and Fibre Location (in Groove/on Surface)

The results of Section 5.1.1 and Section 5.1.2 show that the influence of the ribs cause local variations in the reinforcing bar strains. This section discusses the origin of these variations and the sensors’ performance depending on whether they are glued inside a groove or on the bar’s surface. To this end, a cold-worked reinforcing steel bar with a diameter of 18 mm (specimen cw03) was tested under direct tension. The bar was loaded and unloaded eleven times elastically up to a load of 76 kN and then loaded until failure in the 12th loading cycle. The cross-sections of the reinforcing bar within one complete rib pattern were determined by a surface scan and are shown in Appendix A (Figure A1b).

**Figure 10:**
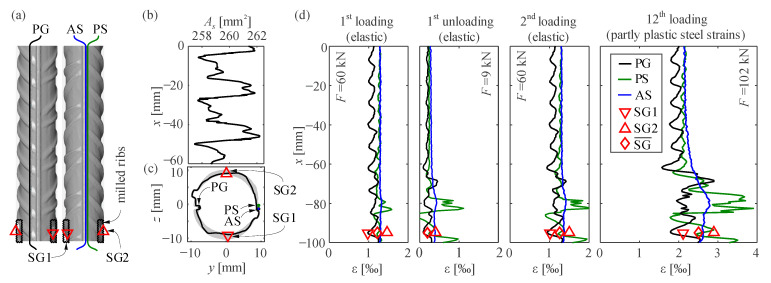
Influence of reinforcing bar geometry and local milling for installation of strain gauges on strain measurements studied on specimen cw03 tested under direct tension: (**a**) Surface scan with instrumentation (SG: strain gauges; PG: polyimide FOS in groove; PS: polyimide FOS on surface; AS: acrylate FOS on surface); (**b**) cross-sectional area distribution; (**c**) bar cross-section and projected total rib area (grey) with instrumentation and (**d**) strain measurements for selected steps (DFOS shows raw data with removed outliers).

Figure 10a shows the 3D surface scan of the bar (front and backside) with the used instrumentation, which consisted of three fibre optical sensors and two strain gauges. Contrary to the recommendation in Section 4.1 of installing the FOS inside a groove not crossing the ribs, the sensors were installed differently in this case:

- PG is a polyimide coated fibre glued with epoxy inside a groove, crossing slightly the ribs.
- An acrylate and a polyimide coated fibre (AS and PS, respectively) were glued with epoxy and thickening agent onto the bar surface along its longitudinal ridge.

One cross-section was instrumented with two strain gauges (SG1 and SG2) of the Kyowa KFD-2-C1-11 (120 ohm, 2 mm) type, at approximately 90° to the fibres (see Figure 10a,c). The section of about 15 mm in which the ribs were milled to ensure a planar surface for gluing the gauges is marked in Figure 10a.

Figure 10d presents the results for four selected steps of the experiment. The analysis of the general behaviour shows different strain results depending on the sensor location over the cross-section, which indicates that the bar experienced a certain amount of bending. The results are strongly disturbed where the ribs were milled (from 70 to −100 mm), where mean strains are higher than in the non-milled region. This might be due to cross-sectional area loss and/or locally altered material properties due to the surface preparation. Besides the differences due to bending, the results of the fibres glued directly on the bar surface were noisier than for the fibre installed in a groove. Moreover, these sensors provided unsteady strain distributions in the milled region even during the elastic cycles (compare 1st and 2nd elastic cycles in Figure 10d). The adhesive peeled off the bar surface at plastic deformations and might have even detached locally for elastic strains given the measured strains did not decrease at some sections much when unloading. These results justify the recommendation of installing the sensors inside a groove, cf. Section 4.1.

The FOS installed inside a groove was the only one able to measure the local strain variations due to ribs. The results of this fibre show that the local strain maxima do not coincide with the maximal cross-sectional area (refer to Figure 10b for the distribution of the cross-sectional area), but rather with the location of ribs and inter-rib spaces. In addition, the variation of cross-sectional area (around 1.5%) is much smaller than the measured strain variations at the ribs (about 15%). Therefore, the strain fluctuations at the ribs should not be attributed to the varying cross-sectional area but to the ribs acting as discontinuities that disrupt the uniaxial strain state in a bar locally.

When investigating the behaviour of cast-in bars, it is crucial to recognise that strain variations due to local load introduction at the ribs from the surrounding concrete (i.e., bond) overlap with the strain variation at the ribs observed in bare bars. Therefore, local bond at single ribs cannot be assessed by simply deriving the raw strain distribution of a bonded bar as proposed by other authors [10].

### 5.2. Measurement of Concrete Compressive Strains

This section presents and discusses the results from a four-point bending test (specimen Nn) in which the compression zone was instrumented with DFOS and a high-resolution 3D-DIC system. The test was part of a larger experimental campaign studying lap splices with conventional and ultra-high performance fibre reinforced concrete [36].

#### 5.2.1. Test Setup and Instrumentation

**Figure 11:**
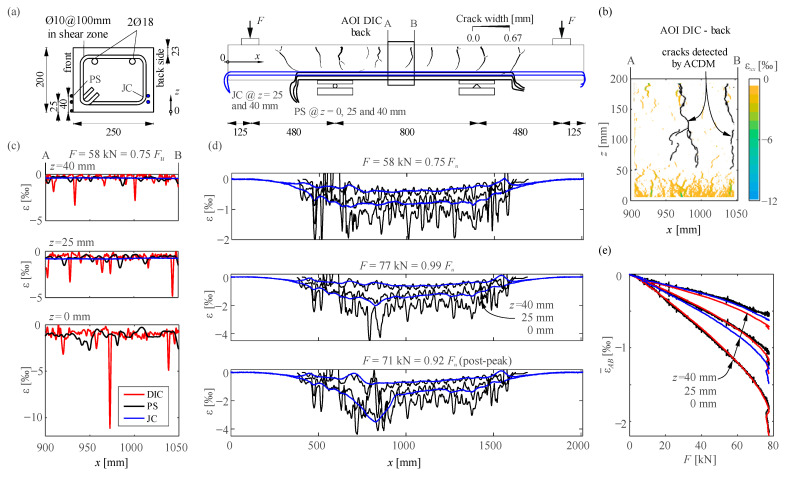
Measurement of concrete compressive strains with DFOS on specimen Nn*:* (**a**) Geometry, test setup and instrumentation (dimensions in mm) with crack pattern and crack widths at *F* = 58 kN(JC: cast jacketed FOS; PS: polyimide FOS on surface); (**b**) DIC strains in the longitudinal direction *x* at *F* = 58 kN; (**c**) strain results along the field of view of DIC at *F* = 58 kN; (**d**) complete DFOS strain distributions at *F* = 58, 77, and 71 kN (post-peak) and (**e**) evolution of average strains within the field of view of DIC.

Figure 11a shows the geometry and reinforcement of the beam, as well as the test setup. The concrete had a compressive cylinder strength of 35.5 MPa, a tensile strength of 2.8 MPa (double-punch test) and an E-Modulus of 30.5 GPa. The reinforcement had a nominal yielding strength of 500 MPa and ductility class B (according to EN 1992-1-1 [37]).

The front side was instrumented with three fibre optical sensors at different heights as shown in Figure 11a. The polyimide coated single-mode fibres (PS) were aligned in *x*-direction, tensioned with about 0.4‰ and then glued with a liquid, fast-setting adhesive at heights *z* = 0, 25, and 40 mm from the bottom edge. Jacketed optical sensors (JC) were also cast in with a 30 mm concrete cover to the backside at *z* = 25 and 40 mm height. The JC sensors were 3.2 mm in diameter and consisted of a central single-mode glass fibre inside a steel tubing coated with polyamide. The polyamide jacket had a ribbed structure for enhanced bond properties. The sensors installation and post-processing of results followed the recommendations given in Section 4.

One 3D-DIC system tracked the displacements of the whole front side of the beam. These displacements were used to determine the global crack pattern at the load *F*= 58 kN shown in Figure 11a with the Automated Crack Detection and Measurement software [26,27]. This paper discusses only the results of a second high-resolution 3D-DIC system on the back. The DIC hardware and correlation software were identical to the ones described in Section 5.1.2 for test *qst02*. The baseline was 350 mm and the stereo angle 20°, resulting in an area of interest (AOI) of 200 × 150 mm (marked in Figure 11a) and an average scale of 0.03 mm/px. The speckle size was 0.18 mm. The correlation was performed using a subset size of 21 px, a step size of 6 px and a strain filter size of 9. Specification of the front 3D-DIC system can be found in the data repository.

#### 5.2.2. Test Results

Figure 11b presents the DIC results of strains in the longitudinal direction *x* at *F* = 58 kN (75% of the ultimate load). At *z* = 0, 25, and 40 mm these strains were averaged over a height of 10 mm and the resulting strain distributions are compared to the raw DFOS data in Figure 11c. The results reveal strain concentrations in the compression zone. The strain concentrations could not be captured with the jacketed sensors, but the average strains over the AOI coincide very well for the three sensors (see Figure 11e). The location of the peaks varies, since the sensors had different locations over the cross-section (see Figure 11a). The peaks of the DIC measurement are more pronounced and coincide very well with the location of cracks in the tension zone (*x* = 910, 970, and 1040 mm on the back and *x* = 855, 975, and 1035 mm on the front). Note that the middle crack formed almost through the entire section, but it was not visible at the front side. The compressive strains between the peaks are much lower and fairly constant in the constant bending zone.

Figure 11d shows the results of DFOS for the entire specimen at *F* = 58 kN, 77 kN, and 71 kN (post-peak) with smoothed data for PS (moving average over 25 data points, i.e., 15.6 mm) and raw data for JC. The results of PS at 0 mm height over the left support are very noisy because the sensor was glued very close to the bearing plate, and local effects might have disturbed the strain field. Except in this area, the local peaks in PS are still distinguishable after data smoothing. Moreover, the strain profiles at various heights have peaks at the same locations. Smaller strain peaks also occur between two cracks, where micro cracks might have formed in the tension zone. The beam failed due to concrete crushing at *x* = 853 mm, where the highest compressive strains were measured (see Figure 11d results for *F* = 77 kN). Concrete spalling prevented the glued polyimide coated fibres and the cast jacketed sensors from properly capturing the post-peak strains at the failure section (correlation was lost after spalling).

#### 5.2.3. Discussion

This study has shown that measuring concrete compressive strains with DIC is very powerful, as it yields full-field strains results of the whole concrete surface. However, measuring strains with a similar sensitivity to DFOS limits DIC’s maximum measuring distance to around 300 mm when using state-of-the-art equipment. Moreover, DIC compressive measurements cannot measure after spalling takes place. Hence, DIC should be seen as an excellent complement to DFOS instrumentation, useful for local measurements or for detecting and measuring cracks.

Fibre optical sensors with jackets cast into the concrete proved to be more robust to measure compressive strains though much less sensitive than the sensors mounted to the surface. They can also capture better the post-peak behaviour since they are less susceptible to superficial spalling effects usually happening during the concrete crushing process. Hence, cast jacketed sensors are preferred to assess mean compressive strains (i.e., for standard applications). When local effects are to be investigated, more sensitive sensors are preferable. Further research is required to analyse the performance of cast in sensors with thinner jackets (e.g., 1 mm in diameter), which could offer robustness at improved sensitivity with respect to the jacket sensors used in this study.

The observed strain concentrations in the compressive zone at the cracks, even for strains of only 1‰, is a novel finding not visible with classical instrumentation technologies. Concrete constitutive relationships have been derived using average strains over a sample length much larger than the strain localisation zone. Moreover, beam cross-sections are typically assumed to remain plane in design, and possible variations of concrete compressive strains between two cracks have rarely been discussed. These new findings on the local behaviour might open the way for the development of new mechanical models that consistently capture the fracture process of concrete.

## 6. From Concrete and Steel Strains to Stresses

The direct use of DFOS strain data is manifold, but for many applications it is required to derive stresses from it. To do so, the constitutive material law should be assumed or, preferably, be measured by material characterisation tests. This section discusses the challenges to estimate in a reliable manner the concrete and reinforcing steel stresses based on the measured strains.

The results from Section 5 already showed potential drawbacks of the high accuracy and resolution of the DFOS measurement. Existing material laws describe and were determined from the mean material behaviour over a certain length, which typically exceeds the virtual gauge lengths used in DFOS by orders of magnitude. Hence, these material constitutive relationships should not be used to derive stresses if the measured strains exhibit local effects that cannot be properly filtered out. For steel, such local strain fluctuations can be due to ribs or material inhomogeneity caused by production processes. For concrete, they may be strain concentrations in the compressive zone, e.g., at cracked cross-sections or due to concrete crushing.

Other limitations arise from the accuracy of the considered constitutive relationships. This limitation applies to any conversion of strains to stresses, irrespective of the used strain measuring technology. While the uniaxial monotonic behaviour of the reinforcement and the concrete can be determined by material characterisation, the following aspects might affect the derivation of stresses in a reinforced concrete structure (non-exhaustive list):

- Cyclic behaviour: The derivation of stresses in a structure requires a constitutive model including the cyclic behaviour. This model is usually not known from the material characterisation. The accuracy of the unloading and reloading branches of existing cyclic models is much lower than for monotonic behaviour. The complexity of the constitutive relationships for cyclic loading has been discussed in Figure 6i that shows that the stiffness of the unloading branches of a reinforcing steel bar changes as a function of plastic deformations. Neglecting this effect for plastic strains of 6‰ might lead to an error of around 15% when estimating the stresses. The modelling of the constitutive law becomes even more challenging for embedded reinforcing bars, since (plastic) strains are highest at the cracked section and decrease with the distance to a crack. Therefore, each section within one crack element undergoes a different load history and may have a different plastic strain and a different constitutive law.
- Stress states different than uniaxial: Stress states of concrete and even reinforcement typically differ from the uniaxial loading applied in material characterisation. Concrete is known to have a constitutive behaviour different from the uniaxial response when confined or even when transversally cracked. Hence, it is challenging to derive concrete compressive stresses except for the compression zone of a plane element. Cast-in bars are subjected to a triaxial stress states due to compressive force introductions at the ribs, which leads to a plastic response different than in the tensile tests of the bare reinforcing bar (this and further issues are discussed in the companion paper [28]).

## 7. Conclusions

This paper summarises the experience with distributed fibre optic strain sensing gained at the Chair of Concrete Structures and Bridge Design at ETH Zurich over the past six years. A concept for the reliable application of DFOS to experimental investigations of structural concrete has been established. The presented best practice of the chair comprises the choice of the suitable sensing fibre, the installation of the glass fibres on the host material, the data acquisition and the post-processing ranging from filtering to the usage of the acquired data. This best practice was developed based on numerous experimental campaigns. Merely a few exemplary tests are presented and discussed within this work.

Distributed fibre optical strain measurements have proven to be a valuable tool in structural concrete research. Nevertheless, the data has to be questioned and used critically since it exceeds the typical resolutions of conventional instrumentation technologies. Existing models were typically calibrated on a global or average behaviour. Hence, DFOS data should be post-processed and filtered properly to eliminate local fluctuations and be able to use it together with existing models. The local information provided by DFOS is still very useful for research purposes as it can provide new insights into the behaviour of reinforced concrete that might lead to improved mechanical models.

A good concept for the instrumentation is crucial for getting meaningful results. The right choice of the fibre optical sensor depends on the measurement goals and may require preliminary tests. A fibre optical sensor with a chemically bonded coating is preferable due to its high sensitivity. Fluctuations in the strain distribution caused by inhomogeneities (material properties, geometry, local deformation concentrations, etc.) can be detected and filtered out in the post-processing if necessary while maintaining the desired resolution. Jacketed fibres might be a good choice for measuring mean concrete compressive strains, as they can be cast inside concrete and are not susceptible to superficial spalling when concrete crushes.

c-OFDR systems can measure beyond their measurement range of typically 15,000 μm/m if new reference states are acquired during testing, but local strain discontinuities above this range could still not be captured. Hence, measuring plastic strains of reinforcing bars with quenched, self-tempered steel (which typically has an extension of the yield plateaus larger than 15,000 μm/m) is challenging. The risk of losing correlation could be minimised and the measurement range increased if the data acquisition systems would implement strain measurements with continuously and automatically updated reference states.

When reinforcing steel bars are instrumented, the most reliable results are obtained by gluing the fibres inside a small longitudinal groove, which does not pass through the ribs. The groove should be planed and not cut into reinforcing steel to minimise heat ingress, which might cause undesired local alterations of material properties. Local bending and clamping should be avoided, e.g., by protecting the fibres ends inside plastic tubes. The results of DFOS measurements revealed that reinforcing bars with cold-worked steel may exhibit locally varying constitutive behaviour, most probably caused by a non-uniform stretching process. These variations lead to local strain variations which might be misinterpreted as cracks when such bars are cast in. Each reinforcing bar should be equipped with at least two fibre optical sensors to identify such behaviour and to enable a meaningful estimation of stresses from the mean measured strain. Local strain variations at the ribs were also observed for all studied steel types and increased proportionally with increasing mean deformations. These variations are not caused by the varying cross-sectional area but by the ribs acting as discontinuities that disrupt the uniaxial strain state in a bar locally. When investigating the behaviour of cast-in bars, it is crucial to recognise that strain variations due to local load introduction at the ribs from the surrounding concrete (i.e., bond) overlap with the strain variation at the ribs observed in bare bars. Therefore, local bond at single ribs cannot be assessed simply by deriving the raw strain distribution of a bonded bar.

The measurements on the compression zone of a reinforced concrete beam subjected to pure bending revealed that the compression strains at the crack sections exceed those between two cracks by an order of magnitude. These local maxima exhibit very narrow, sharp peaks, indicating that reinforced concrete segments between adjacent bending cracks behave almost as rigid bodies, with deformations and curvatures localising at the crack sections already at moderate load levels. This behaviour has not been reported beforehand to the authors’ knowledge, possibly due to the limitations of the instrumentation technologies used in the past. This and new findings of DFOS on the local behaviour might open the way for the development of refined and more consistent structural concrete models.

## Figures and Tables

**Table 1:** Overview of the experiments used for the basic investigations.

Section	Specimen		Loading	Investigations
Section 5.1.1	cw01	Cold-worked ribbed steel bar, Ø*_s_*= 14 mm	Uniaxial tension	Effect of ribs Comparison to LVDTs
Section 5.1.1	cw02	Cold-worked ribbed steel bar, Ø*_s_*= 20 mm		Influence of the cold-working process
Section 5.1.2	qst01	Quenched and self-tempered ribbed steel bar, Ø*_s_*= 20 mm		Steel yielding (Lüders bands)
Section 5.1.2	qst02	Quenched and self-tempered ribbed steel bar, Ø*_s_*= 26 mm		Propagation of Lüders bands based on 3D-DIC measurements (no DFOS)
Section 5.1.3	cw03	Quenched and self-tempered ribbed steel bar, Ø*_s_*= 18 mm		Influence of the ribs|Comparison of FOS types Comparison to strain gauges|Bar surface scan
Section 5.2	Nn	Reinforced concrete beam, C25/30	Pure bending	Comparison of FOS types Comparison to 3D-DIC measurement

## Data Availability

The data presented in this study are openly available in ETH Research Collection at DOI:10.3929/ethz-b-000504568.

## Appendix A

A surface scan was made of specimens cw01 and cw03. Figure A1 presents ten equally spaced sections of both bars ranging over one repeating rib pattern.

## References

[1] Iten M. (2012). Novel Applications of Distributed Fiber-Optic Sensing in Geotechnical Engineering.

[2] Hauswirth D. (2014). A Study of the Novel Approaches to Soil Displacement Monitoring Using Distributed Fiber Optic Strain Sensing. Ph.D. Thesis.

[3] Monsberger C.M., Lienhart W. (2019). Design, Testing, and Realization of a Distributed Fiber Optic Monitoring System to Assess Bending Characteristics Along Grouted Anchors. J. Lightwave Technol..

[4] Lienhart W. (2015). Case Studies of High-Sensitivity Monitoring of Natural and Engineered Slopes. J. Rock Mech. Geotech. Eng..

[5] Eickhoff W. (1981). Optical Frequency Domain Reflectometry in Single-Mode Fiber. Appl. Phys. Lett..

[6] Glombitza U., Brinkmeyer E. (1993). Coherent Frequency-Domain Reflectometry for Characterization of Single-Mode Integrated-Optical Waveguides. J. Lightwave Technol..

[7] Samiec D. (2011). Verteilte Faseroptische Temperatur-Und Dehnungsmessung Mit Sehr Hoher Ortsauflösung. Photonik.

[8] Betschoga C.T., Monsberger C. (2018). Bestimmung des Kraftflusses anhand verfeinerter Messmethoden bei Querkraftversuchen an Stahlbetonbalken. Proceedings of the 4. Grazer Betonkolloquium.

[9] Malek A., Scott A., Pampanin S., Hoult N.A. (2019). Postyield Bond Deterioration and Damage Assessment of RC Beams Using Distributed Fiber-Optic Strain Sensing System. J. Struct. Eng..

[10] Cantone R., Ruiz M.F., Muttoni A. (2020). A Detailed View on the Rebar–to–Concrete Interaction Based on Refined Measurement Techniques. Eng. Struct..

[11] Klug F., Lackner S., Lienhart W. Monitoring of Railway Deformations Using Distributed Fiber Optic Sensors. Proceedings of the Joint International Symposium on Deformation Monitoring.

[12] Quiertant M., Baby F., Khadour A., Marchand P., Rivillon P., Billo J., Lapeyrere R., Toutlemonde F., Simon A., Cordier J. (2012). Deformation Monitoring of Reinforcement Bars with a Distributed Fiber Optic Sensor for the SHM of Reinforced Concrete Structures.

[13] Schmidt-Thrö D.-I.G., Scheufler D.-I.W., Fischer O. (2016). Kontinuierliche faseroptische Dehnungsmessung im Stahlbetonbau. Beton-und Stahlbetonbau.

[14] Mata-Falcón J., Haefliger S., Lee M., Galkovski T., Gehri N. (2020). Combined Application of Distributed Fibre Optical and Digital Image Correlation Measurements to Structural Concrete Experiments. Eng. Struct..

[15] Brault A., Hoult N. (2019). Distributed Reinforcement Strains: Measurement and Application. ACI Struct. J..

[16] Monsberger C.M., Lienhart W. (2021). Distributed Fiber Optic Shape Sensing of Concrete Structures. Sensors.

[17] Kenel A. (2002). Biegetragverhalten und Mindestbewehrung von Stahlbetonbauteilen.

[18] Brault A., Hoult N. (2019). Monitoring Reinforced Concrete Serviceability Performance Using Fiber Optic Sensors. ACI Struct. J..

[19] Fischer O., Thoma S., Crepaz S. (2019). Distributed Fiber Optic Sensing for Crack Detection in Concrete Structures. Civ. Eng. Des..

[20] Lemcherreq Y., Kaufmann W., Vogel T. Fatigue of Bond: Experimental Investigation Using Pull-Out Tests with Distributed Fibre Optical Sensors. Proceedings of the Fib Symposium: Concrete Structures for Resilient Society (Fib 2020) (virtual).

[21] Galkovski T., Mata-Falcón J., Kaufmann W. Determination of the Effective Concrete Area in Tension Relevant for Modelling Tension Stiffening in SLS and ULS Design. Proceedings of the Fib Symposium 2021.

[22] Karagiannis D. (2021). Effect of Transverse Bending on the Shear Capacity of Concrete bridges. Ph.D. Thesis.

[23] Beck A. (2021). Paradigms of Shear in Structural Concrete: Theoretical and Experimental Investigation. Ph.D. Thesis.

[24] Zhang S., Liu H., Cheng J., DeJong M.J. (2020). A Mechanical Model to Interpret Distributed Fiber Optic Strain Measurement at Displacement Discontinuities. Struct. Health Monit..

[25] Billon A., Hénault J.-M., Quiertant M., Taillade F., Khadour A., Martin R.-P., Benzarti K. (2015). Qualification of a Distributed Optical Fiber Sensor Bonded to the Surface of a Concrete Structure: A Methodology to Obtain Quantitative Strain Measurements. Smart Mater. Struct..

[26] Gehri N., Mata-Falcón J., Kaufmann W. (2020). Automated Crack Detection and Measurement Based on Digital Image Correlation. Constr. Build. Mater..

[27] Gehri N., Mata-Falcón J., Kaufmann W. (2021). Refined Extraction of Crack Characteristics in Large-Scale Concrete Experiments Based on Digital Image Correlation. Eng. Struct..

[28] Lemcherreq Y., Galkovski T., Mata-Falcón J., Kaufmann W. (2021). Application of Distributed Fibre Optical Sensing in Reinforced Concrete Elements Subjected to Monotonic and Cyclic Loadings. Sensors.

[29] Hoepffner R. (2008). Distributed Fiber Optic Strain Sensing in Hydraulic Concrete and Earth Structures: Measuring Theory and Field Investigations on Dams and Landslides.

[30] LUNA (2021). ODiSI 6000 Data Sheet. Luna Innovations Incorporate. https://lunainc.com/sites/default/files/assets/files/data-sheet/LUNA%20ODiSI%206000%20Data%20Sheet.pdf.

[31] Haefliger S., Kaufmann W. (2021). Influence of Cross Section Loss on the Stress-Strain Characteristics of Corroded Quenched and Self-Tempered Reinforcing Bars. Constr. Build. Mater..

[32] Evers J. (2020). Untersuchungen zur Messunsicherheit Faseroptischer Dehnungsmessungen in Stahlbetonelementen mit dem ODiSI-6100. Master’s Project Thesis.

[33] Pantazopoulou S.J., Tastani S.P., Thermou G.E., Triantafillou T., Monti G., Bournas D., Guadagnini M. (2016). Structural Concrete. Struct. Concr..

[34] Fahrni R., Reist F. (2015). Evaluierung Von Messsystemen für Ausziehversuche an Einbetonierten Bewehrungsstäben. Master’s Thesis.

[35] Correlated Solutions (2019). Vic-3D Software Manual.

[36] Wyss J. (2021). Global and Local Analysis of UHPFRC Lap Splices with Refined Distributed Optical Measurements. Master’s Thesis.

[37] European Committee for Standardization (2004). Eurocode 2: ‘Design of Concrete Structures—Part 1-1: General Rules and Rules for Buildings (EN 1992-1-1)’.

